# Fractal-Cluster Theory and Its Applications for the Description of Biological Organisms

**DOI:** 10.3390/e25101433

**Published:** 2023-10-10

**Authors:** Vyacheslav Theodorovich Volov

**Affiliations:** Natural Science Department, Samara State University of Railway Transport, Perviy Bezimyaniy Pereulok 18, 443066 Samara, Russia; volovvt@mail.ru

**Keywords:** biological organism, fractal-cluster theory, fractal-cluster entropy, fractal-cluster criteria, fractal-cluster stochastics, evolution laws

## Abstract

This article presents an overview of an alternative approach to the systematization and evolution of biological organisms on the basis of the fractal-cluster theory. It presents the foundations of the fractal-cluster theory for the self-organizing systems of the organism class. Static and dynamic efficiency criteria based on the fractal-cluster relations and the analytical apparatus of nonequilibrium thermodynamics are presented. We introduce a highly sensitive static criterion, *D*, which determines the deviation in the value of the clusters and subclusters of the fractal-cluster system structures from their reference values. Other static criteria are the fractal-cluster entropy *H* and the free energy *F* of an organism. The dynamic criterion is based on Prigogine’s theorem and is determined by the second differential of the temporal trend of the fractal-cluster entropy *H*. By using simulations of the cluster variations for biological organisms in the (*H*, *D*, *F*)-space, the criteria for the fractal-cluster stochastics as well as for energy and evolution laws are obtained. The relationship between the traditional and fractal-cluster approaches for identifying an organism is discussed.

## 1. Introduction

The history of the emergence of the fractal-cluster approach to the study of biological organisms goes back to the 1960s of the last century. It was at that time that Academician S.P. Korolev, General Director of the famous firm “Energia” (the flagship of Soviet cosmonautics), assigned the following task to his young colleague, V.P. Burdakov. It was necessary to determine the optimum ratio between the rocket’s resources related to its power, transportation, protection, technological and control units. The solution to this problem required, in turn, a general analysis of the optimum correlation between resources for various complex self-organizing systems (SOS). To quantitatively describe the resources of such systems, V. P. Burdakov used extensive parameters of these systems (mass, volume, time, etc.). First of all, he analyzed the best Russian and American rockets, which were independently created by a number of scientists and engineers from different countries. Of course, rockets are the most complex technical systems which can be considered as self-organizing systems in the “man–technical system” class. The shares of resource (mass) of a rocket which refer to energy, transport, security, technology and control subsystems were determined. Later, when analyzing organismal systems, the security and control subsystems were renamed into the ecology and information subsystems. These shares in the best Russian and American rockets turned out to be approximately equal. In addition, the corresponding resource distributions of various technical systems (the “man–technical system” class) were also studied. The next step of his investigation was the study of biological organisms, which were created by nature as a result of evolution. He determined the relations of the mass shares of organisms (39 organism species starting with Chlamydomonas and ending with whales) with their energy, transport, ecology, technology and information needs. These basic needs have been identified as corresponding clusters. The proportion of the organism’s resources corresponding to the technology cluster in the application to any biological organism should be interpreted as the share of the organism’s resources that allows for performing the functions of the organism aimed at realizing its energy and protective needs. For biological organisms, the term “technology cluster” can be renamed to “transformation cluster”. The results of V.P. Burdakov’s research are most fully reflected in monographs [1,2] (unfortunately, only in Russian editions).

We will number the basic needs with an index *i* (the values i=1,2,3,4,5 correspond to the energy, transport, ecology, technology and information needs, respectively). We will also denote by $C_{i}$ the value of any extensive parameter (mass, time, etc.) that belongs to the cluster (basic need) with the number *i*. The $C_{i}$ unit is the same as that of its extensive parameter (mass, time, etc.). The ${\bar{C}}_{i}$ values (with a bar) are normalized and are measured in fractions (or percentages) of the extensive parameter for the whole organism. A statistical analysis of biological organisms allowed us to obtain the ideal distribution in clusters. The corresponding reference (ideal) values ${\bar{C}}_{i}$ are determined in fractions of the total resource 38%$\left(0.38\pm 0.06\right)$, 27%$\left(0.27\pm 0.05\right)$, 16%$\left(0.16\pm 0.04\right)$, 13%$\left(0.13\pm 0.02\right)$ and 6%$\left(0.06\pm 0.01\right)$ for i=1,2,3,4,5, respectively, that is, they are dimensionless quantities. In the context of the study of a biological organism, the mass of the organism is taken as an extensive parameter of 100%, and the corresponding fractions of the organism mass are 0.38, 0.27, 0.16, 0.13 and 0.06 and correspond to the amount of resources in the energy, transport, ecology, technology and information systems of the organism. The sum of the five clusters expressed in units of the system’s resources, representing any extensive parameter (mass, time, etc.), is a constant value (100%) at a certain interval of the organism life.

**Figure 1:**
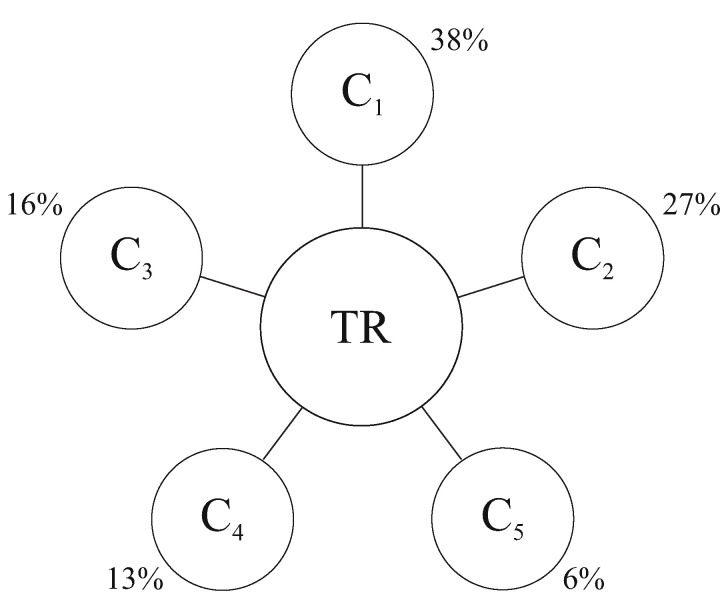
Fractal-cluster scheme of the organism’s resource distribution (one-level case); TR—an organism’s total resource. The percentages in the figure are the reference values of the clusters of the organism.

**Figure 2:**
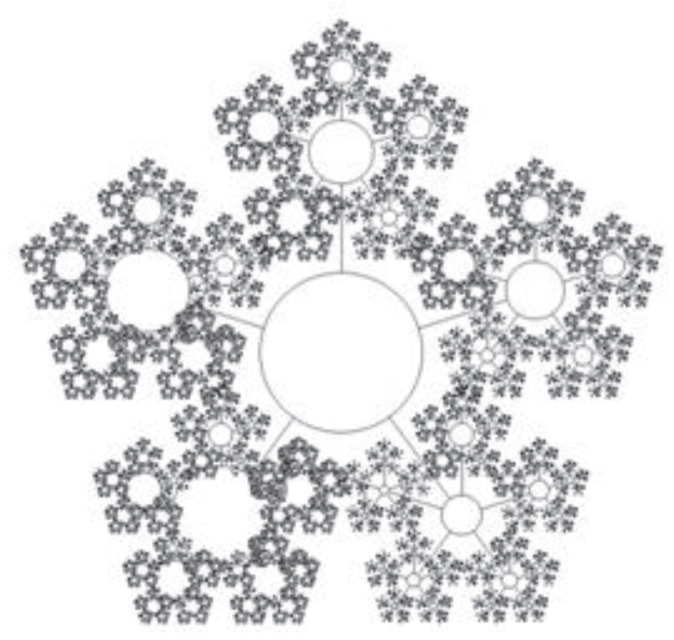
Schematic of the SOS’s resource allocation for a hypothetical 6-level fractal-cluster system. The figure is a tree graph with the 6 hierarchical levels and circled vertices, each circle being a cluster of the corresponding hierarchy level.

For a number of systems belonging to the class of organism (this term will be explained in the next chapter in detail), each cluster can be a functioning subsystem in which basic needs can also be identified, similar to those that can be identified in the organism. However, the cluster of an organism cannot be considered as a separate organism, because the cluster cannot function separately from the organism without violating its integrity. We can divide each cluster into five subsystems—subclusters—each of which has its own basic function, coinciding with one of the basic needs of the entire system. For example, the resource in the energy cluster can be shared for supporting its own energy, transport, ecology, technology and information subclusters. It can be expected that such a partition can be extended to subclusters of the *n*-th level. However, for real systems, it is very problematic to identify subclusters of levels greater than n=2 because it requires a separate study. For biological organisms, as a rule, two-level clustering (n=2) is sufficient because the number of hierarchy levels in real systems is always finite and, as a rule, in a real analysis, the number of levels that can be identified does not exceed n=3. Namely, each of the subclusters of a given level can be considered as the union of the five higher-level subclusters. Such systems are called the fractal-cluster (FC) systems (see [1,2,3,4]). Thus, the space of the resource distribution for the same systems has a hierarchical structure which can be described with a *n*-level hierarchical tree with a fixed number p=5 of branches. Figure 1 and Figure 2 represent an illustration of the resource distribution of an organism at one (Figure 1) and six levels (Figure 2) of the hierarchy of resource distribution in the $5^{n}$-dimensional FC space. Figure 2 corresponds to the idealized case and is a tree graph, with six hierarchy levels and with the vertices denoted by circles, each circle being a cluster of the corresponding hierarchy level.

It should be emphasized that the fractal-cluster description of an organism exclusively refers to the distribution of its resource according to its basic needs.

The closest values of clusters to their ideal values were obtained for young men under 25 years of age [1]. These ideal (standard) relations among the clusters were named the fractal-cluster relations (FCR). In the fractal-cluster approach, the normal (effective) functioning of an organism is understood as such functioning in which the mathematical expectation of cluster values are close to the ideal (reference) values of clusters. For the organism with normal functioning of any nature (biological, social and economical), these ideal values have approximately the same values. A natural question arises—why are exactly the five basic needs involved when defining such a concept as an organism in the context of our approach? These needs (energy, transport, ecology (or security), technology (or transformation) and information (or management) are basic, because without their independent satisfaction (without outside help) the organism cannot exist. From the point of view of the fractal-cluster approach, an organism can be considered any self-organizing system (SOS) in which the five basic needs can be identified that are satisfied by the SOS, independently. However, one can note that the number five also occurs in various areas of the natural sciences and is also a quantitative measure of the properties of the certain biological systems. For example, it is known that the thermodynamics of the stable states have five thermodynamic potentials [5]. Humans have five sense organs, five brain rhythms, and in human anatomy the number five is highlighted [6,7]. When considering flowers, when the number of petals is a multiple of five, there is always an ovary; otherwise, there is no ovary. Other examples can be given, but of course this is not scientific justification for choosing the number five as the number of clusters. In fact, the offered FC approach is qualitatively different from the traditional approach for the description of a complex SOS. The offered approach [1] was based on the fractal structure basis—the FC matrix—allowing actively to describe the resource distributions in the SOS. It must be emphasized that, in contrast to the fundamental works of Benoit Mandelbrot [8], Per Bak [9,10] and other researchers, fractals are observable objects (directly in nature or in computational models), with the fractal-cluster approach to describing a self-organizing system (organism) its system-forming element (fractal-cluster matrix of system resources), a construct (model) similar in structure to an *n*-level fractal, which is intended for the study of self-organizing systems of the specified class. Unfortunately, both theory and mathematical models on the basis of this approach have not been developed. That is why, in 1998, Professor V. P. Burdakov offered his young colleague (the author of this article) to start developing the fractal-cluster theory.

The little fame of the offered approach in the broader scientific community is explained by the fact that publications did only appear in the Russian language. The aim of this article is, on the one hand, getting biological scientists acquainted with the offered FC approach and, on the other hand, exposition of the new results obtained in the development of this approach. The possibility of using the FC models for the resource distribution analysis is based on a range of research [1,2,3,4], in which the research of the optimal resource distribution is presented. These methods are based on the thermodynamic method in its informational interpretation. Now, one can show some conformations of the validity of the FC structuring for the SOS. These confirmations are the following: (1) the set of experimental biological data is analyzed by mathematical statistical methods [1]; and (2) the theoretical value of the transport cluster is obtained by using the limit energy theorem for gas flow systems [11].

This paper is organized as follows. In Section 2, the features of identification of the term “organism” with the fractal-cluster approach to research in biology are presented. Section 3 is devoted to the FC criteria of the fractal-cluster theory. An analysis of the static and dynamic stability for the “organism” evolution are presented in Section 4. The stochastic distributions in FC theory are considered in Section 5. A discussion and some applications of FC theory for biological organisms and prospects for further research are discussed in Section 6.

## 2. Features of Identification of the Term “Organism” in the Fractal-Cluster Approach to Biology Research

As noted above, in the fractal-cluster approach, the “organism” is understood as a complex SOS in which the five basic needs can be identified: energy, transport, ecology, technology and information needs. These needs are called clusters that are named accordingly (energy cluster, etc.). It should be noted that such names of the clusters have a historical imprint, and this is due to the fact that the initial classification of resources was not for biological organisms but in technological systems. As a rule, we will use the same names for clusters of biological systems. The technology cluster can also be called a transformation cluster. Note also that there are many more needs for the self-organizing systems, but all of them can be “packaged” in the hierarchical fractal structure, with the self-similar subclusters of *n*-levels. As a resource, any extensive parameter of an organism (mass, time, volume, etc.) is used. From the point of view of the fractal-cluster approach, any complex system in which it is impossible to distinguish the five basic needs is not an organism, and the announced approach cannot be applied to it.

It must be emphasized that resource distribution in the FC systems is a nontrivial task which requires special skills and study. For example, when choosing the organism mass as an extensive parameter, a separate organism’s structure can perform several functions and therefore belong to different clusters. According to the general methodological approach adopted in the fractal-cluster theory of attributing a part of an organism to any of its subsystems, the clusterization of an organism’s resource is determined by its objective function in this organism. To explain this concept, we will give some examples. For example, if the mass of the human body is considered as a resource, then the mass of the legs and part of the muscular apparatus belongs to the transport cluster. Of course, some people are able to walk on their hands for a while, but this does not mean that hands can be attributed to a transport cluster. On the contrary, monkeys use all four limbs for movement and activity (searching for food, etc.), i.e., the mass of limbs and parts of the muscular apparatus belong to two clusters: transport and technological. In some species of monkeys, the tail can also perform a transport function. However, determining the ratio between the shares of resources in these animal clusters, i.e., the implementation of the fractal-cluster analysis, requires structuring the temporary resource of the animal for a certain long period of time.

An example of the most developed self-organizing system among the biological systems considered is the human organism. For this system as the “resource”, we can choose a share of time on a certain time scale (for example, a period of 24 h) needed for satisfying the corresponding five basic needs. Bearing it in mind, a time share spent on satisfying energy needs (sleep, food intake, etc.) is included in the energy cluster. The transport cluster consists of a share of time spent on transfer. A share of time spent on rest, wellness treatments, etc., is included in the ecology cluster. Working time represents the share of resources going to the technology cluster. The information cluster includes the time spent on obtaining new knowledge and skills. We have to emphasize that for a healthy individual (18–64 years old), according to the National Sleep Foundation [12], sleep time is 7–9 h. Based on statistical data, it was shown in [13] that meal time (without talking) should not be less than 20 min, that is, with three meals a day, it is one hour. Thus, the ratio of sleep and meal times to 24 h is close to the value of the ideal value of the energy cluster (8/24≈0.38), etc.

According to the fractal-cluster approach, all movements should take approximately 6.5 h; rest takes 3.5 h, technology or activity is about 3 h and the effective receipt and processing of information takes about 1.5 h. These assessments are correlated with the World Health Organization guidelines [14]. If you use the organism’s weight (of a young person) as an extensive parameter, then, for example, the transport cluster is the ratio of the weight of the legs and part of the muscle apparatus to the weight of the entire body. The ratio will be close on average to 0.27, i.e., it is approximately equal to the ideal value of the transport cluster [1,2,3,4]. The technology cluster, a ratio of the weight of the arms and part of the muscle apparatus to the weight of the entire body, will be close to the ideal value of the technology cluster, 0.13. This ratio of the energy cluster including the respiratory, digestive, fat and circulatory systems will be close on average to 0.38. The information cluster ratio which includes the brain, sexual system and nervous system will be close on average to 0.06. It should be noted that a prolonged significant deviation in the resource values in the clusters from their ideal (reference) values leads to a disturbance of the organism’s work accompanying various pathologies. The necessary and sufficient condition of the definition of the term “organism” on the basis of the fractal-cluster approach should be highlighted. The necessary condition is the presence in a complex system of the five above-mentioned basic needs. A sufficient condition is the ability of the organism itself to satisfy its basic needs. Thus, for example, from the point of view of the fractal-cluster theory, a baby, despite the presence of five basic needs, is not an organism because, without the mother’s help, they cannot move, receive food, etc. The definition of the organism in the fractal-cluster approach is qualitatively different from the generally accepted one in biology and biophysics. For example, classical factors of determining an organism [15], the (1) cell structure, (2) reproduction, (3) growth and development, (4) energy utilization, (5) response to the environment, (6) homeostasis and (7) evolutionary adaptation, determine mainly the external characteristics and results of the life activity of an organism interacting with an external environment. The fractal-cluster approach in the study of biological organisms determines the distribution of the resource within an organism according to its basic needs (clusters) and it does not determine the growth of an organism. However, the growth factor of an organism is related to changes in the total resource of the organism. To overcome this, the resource–change curve is divided into a number of sections in which the change in the organism’s resource can be neglected (quasi-homeostasis). In this case, at each such stage of the development of the organism, the criteria apparatus of the FC theory is applicable. This approach is similar to the classical thermodynamic method, when the nonequilibrium process is replaced by a series of equilibrium processes when the initial and the final states of the process coincide. The energy utilization factor (4) undoubtedly correlates with the energy cluster of the fractal-cluster approach to organism studies. Factors (5)–(7) correlate with the clusters of ecology (safety), information (controlling) and technology (transformation). Environmental impacts, such as a lack of food, or internal causes (an organism’s disease) lead to a change in the distribution of resources in the corresponding clusters. The organism, having received information about the imbalance of resources (information cluster), carries out a transformation of resources (technology cluster), which in turn leads to a state of sustainable equilibrium with an environment, i.e., homeostasis. The homeostasis factor correlates with the oscillations of clusters and fractal-cluster values near their standard (reference) values. A cellularly ordered structure in a certain sense corresponds with the fractal-cluster hierarchical structure of the organism’s resource representation. Thus, it is possible to summarize that, despite the difference to the classical organism’s definition, the introduced concept of the organism in the fractal-cluster approach does not contradict the classical representation of an organism in biology, on the one hand, and allows us to develop a new toolkit for the study of organism development, on the other hand (Table 1).

## 3. Criteria of the Fractal-Cluster Theory

This section presents the FC criteria of the resource distribution for organisms based on the synthesis of the FCR and nonequilibrium thermodynamics. This resource research of organisms is based on the FC approach and shows that the researched object in question is not decomposed but is a “black box”, which corresponds to the principles and methodology of thermodynamics. In the physical space, the real object has the resources $X_{i}$, necessary for its functioning, and the results of its activities. While transferring the external variables from the physical space to the $5^{n}$-dimensional FC space, the decomposition and classification of information about the object resources have been accomplished, i.e., the FC structurization of the information about necessary resources for organisms (energy, transport, ecology, technology and information support services). As shown in [3,4], thermodynamic laws and theorems make it possible to analyze the stability and the resource allocation efficiency of organisms, without additional empirical information.

The FC criteria of an organism are defined in a nontrivial way with the construction of the fractal-cluster model. In the FC space, the cluster values $\left\{C_{i}\right\}$ and *n*-level subcluster values $\left\{C_{i_{1}i_{2}\cdots i_{n}}\right\}$ for any *n* are positive numbers. As defined above, the unit is the same as the extensive parameter (mass, time, etc.), see Section 1. Further, it is convenient to enter the normalized values of the resources in the clusters:

$${\bar{C}}_{i_{1}i_{2}\cdots i_{n}}=\frac{C_{i_{1}i_{2}\cdots i_{n}}}{C_{\Sigma }},\;C_{\Sigma }=\sum_{i=1}^{5}C_{i}.$$

The values ${\bar{C}}_{i_{1}i_{2}\cdots i_{n}}$ form an *n*-dimensional matrix, which we will call the resource allocation matrix or the fractal-cluster matrix (FCM). The permissible clusters value scope is defined as follows,

(1) $$0<{\bar{C}}_{i}<a_{i},\;\mathrm{and}\;0\leq {\bar{C}}_{i_{1}i_{2}\cdots i_{n}}<a_{i_{1}i_{2}\cdots i_{n}}\;\mathrm{for}\;\mathrm{all}\;n\geq 1,$$

where $0<a_{i_{1}i_{2}\cdots i_{n}}<1$ and conservation law holds:

(2) $$\sum_{i_{1}=1}^{5}\sum_{i_{2}=1}^{5}\ldots \sum_{i_{n}=1}^{5}{\bar{C}}_{i_{1}i_{2}\cdots i_{n}}=1.$$

Equation (1) determines the range of changes in the value of the clusters and subclusters of the organism [1,2,3,4]: their positivity for n=1 ($0<{\bar{C}}_{i}$), non-negativity for n>1, ($0\leq {\bar{C}}_{i_{1}i_{2}\cdots i_{n}}$) and their limitedness (${\bar{C}}_{i_{1}i_{2}\cdots i_{n}}<a_{i_{1}i_{2}\cdots i_{n}}$, where $a_{i_{1}i_{2}\cdots i_{n}}<1$) for all *n*. Equation (2) determines the law of conservation of the organism’s resource at a given stage of the organism’s development. Section 5 presents an algorithm for determining the coefficients $a_{i_{1}i_{2}\cdots i_{n}}$, based on the possible values of the cluster combinations and the conservation law (2).

The FC criteria are based on the following:

(1) The axiom of the FCR universality (the organism’s five-cluster structuring of the resource needs);
(2) The assumption that clusters $\left\{{\bar{C}}_{i}\right\}$ cannot take zero values: ${\bar{C}}_{i}>0;$
(3) The assumption that an organism’s effective functioning in the physical space corresponds to the effective functioning in the FC space.

Initially, the FC approach which has been proposed in [1] was based on intuitive concepts and analogies and then on hard concepts [3]. In connection with the above, it is logical to formulate a criterion for the efficiency of the FC matrix on the fundamental principles and methods of the thermodynamics of stable states.

One of the most important quantities in FC theory is FC entropy, which is an energy function of resource distribution. The formal definition of this function is as follows. To determine the FC entropy *S* for a general *n*-level system, we will impose the following requirements on it [16]:

(1) Additivity;
(2) Positive definiteness;
(3) Finiteness.

In addition, we will require the following:

(4) The FC entropy depends only on the resource values in the energy cluster as well as the resource values in the higher-level energy subclusters of all non-energy clusters.

Requirement (1) is satisfied if *S* is a linear homogeneous function of the resource values in the energy cluster and in the higher-level energy subclusters of the non-energy clusters. Then, requirements (2) and (3) are satisfied if a simple sum of the resource values is chosen as such a linear function. Given requirement (4), this leads to the following expression for *S*:

$$S=C_{1}+\sum_{i_{1}=2}^{5}\left(\sum_{i_{2}=1}^{5}\ldots \sum_{i_{n-1}=1}^{5}C_{i_{1}i_{2}\ldots i_{n-1}1}\right).$$

Because *S* is finite, it is convenient to work with the normalized entropy *H* instead, which is *S* divided by the value of the total resource $C_{\Sigma }$:

(3) $$H=\frac{S}{C_{\Sigma }}={\bar{C}}_{1}+\sum_{i_{1}=2}^{5}\left(\sum_{i_{2}=1}^{5}\ldots \sum_{i_{n-1}=1}^{5}{\bar{C}}_{i_{1}i_{2}\ldots i_{n-1}1}\right),$$

We will call this value the normalized *n*-level fractal-cluster entropy and denote it also by $H_{n}$. The proposed expression for FC entropy has the following meaning: it is the proportion of the organism’s resources used to satisfy all its energy needs in all clusters.

It is necessary to clarify that the proposed mathematical measure is a fractal-cluster entropy that differs significantly from the usual forms of entropy recording, which have a logarithmic deterministic or probabilistic form. However, the basic characteristics of fractal-cluster entropy completely correspond to the properties of the construction of this function [16]. An example of the use in physics of a non-logarithmic form of entropy is the entropy of black holes introduced by J.D. Bekenstein, which is proportional to the area of the event horizon [17].

Let us consider the matrix of the FC ideal states (two-dimensional case n=2, Table 2). The first column numbers the rows of the matrix. In this case, the line numbers i=1,2,3,4,5 correspond to the numbers of the clusters (1—energy, 2—transport, 3—ecology, 4—technology and 5—information). The second column of the *i*-th row contains the ideal values of these clusters. Each cluster is divided into subclusters, and the 3, 4, 5, 6 and 7 columns of the *i*-th row contain the ideal values of the subclusters of the *i*-th cluster. The sum of the values of the subclusters of the first row and the first column of the resources of the ideal matrix give quantitative information about the total share of the organism’s energy resources, which is equal to 0.615. This number is very close to the so-called “Golden Ratio” 0.618, known from numerous publications as the basis of the beauty and harmony of both natural and anthropogenic phenomena [18,19].

The ideal FCM is symmetric for the reference values. The elements of the matrix are ${\bar{C}}_{ij}^{ideal}={\bar{C}}_{i}^{ideal}{\bar{C}}_{j}^{ideal}$. At the same time, $\sum_{j=1}^{5}{\bar{C}}_{ij}^{ideal}={\bar{C}}_{i}^{ideal}.$ The total share of the organism’s energy resources being the major determinant of the organism’s functioning effectiveness is determined by the following formula:

(4) $${\bar{C}}_{\Sigma }^{energy}=\sum_{j=1}^{5}{\bar{C}}_{1j}^{ideal}+\sum_{i=2}^{5}{\bar{C}}_{i1}^{ideal}={\bar{C}}_{1}^{ideal}+{\bar{C}}_{1}^{ideal}\sum_{i=2}^{5}{\bar{C}}_{i}^{ideal}=\;={\bar{C}}_{1}^{ideal}+{\bar{C}}_{1}^{ideal}\left(1-{\bar{C}}_{1}^{ideal}\right)=2{\bar{C}}_{1}^{ideal}-{\left({\bar{C}}_{1}^{ideal}\right)}^{2}=0.615\approx H_{0},$$

where *H*_0_≡ 0.618. The expression (4) is nothing but the entropy of a 2-level FCM with an ideal distribution of resources. This formula presents the resource’s distribution of an organism obtained in [1,4]. For the nonideal distribution of the organism resources, the fractal-cluster entropy for the two-dimensional FCM has the following form:

(5) $$H_{2}={\bar{C}}_{1}+\sum_{j=2}^{5}{\bar{C}}_{j1}.$$

The relationship among the FCM elements for the ideal and nonideal cases and the fractal-cluster entropy $H_{2}$ noted above allows us to find a solution to the FCM for the purpose of optimal evolution ${\bar{C}}_{1j}={\bar{C}}_{1j}\left(t\right)$ (j=1,…,5) and ${\bar{C}}_{i1}={\bar{C}}_{i1}\left(t\right)$ (i=2,…,5), from the nonideal state of the organism (a nonideal FCM) to the perfect condition (an ideal FCM), so that the sum of the FCM elements of the first column and the first row (4) goes into their ideal value, that is, the “Golden Ratio” entropy value is achieved: $H_{2}\left(\left\{{\bar{C}}_{ij}\left(t\right)\right\}\right)\to H_{0}$.

The above proposed criterion of the FC entropy *H* (3) can be attributed to the static criteria. In addition, the criteria of full effectiveness $\eta^{\Sigma }$, proposed in [1], can be treated as the static criteria of the organism’s full effectiveness. The full effectiveness $\eta^{\Sigma }$ of an organism is defined as the minimum ratio $\frac{C_{i}}{C_{i}^{ideal}}$, so named Libih’s barrel [1]:

(6) $$\eta^{\Sigma }=\min_{i}\left(\frac{C_{i}}{C_{i}^{ideal}}\right).$$

Equation (6) is a criterion for the efficiency of the life of an organism, introduced in [1].

To estimate the maximum work performed by the system, we use the thermodynamic potential of the system *F* [3], defined as

F=U−TS,

where *U* is the internal energy of the system, *T* is the temperature and *S* is the thermodynamic entropy. As it is known, this potential is called the free energy. This potential characterizes the maximum possible work that the organism can perform.

An analogue of the potential *F* (Gibbs potential) in the fractal-cluster models [3,4] is the free fractal-cluster energy of the organism defined by the following expression:

(7) $$F=C_{1}-H.$$

But these criteria $\left(H,\;\eta^{\Sigma },\;F\right)$ are not sensitive enough, that is, with small changes in the clusters, small changes in the criteria take place. This fact does not allow for predicting in advance the crisis tendencies of the functioning of the organism.

To determine a highly sensitive criterion of the organism’s resource distribution, Hausdorff’s approach is used. In contrast to the purely fractal structures, the FC *n*-dimensional matrix substantially differs from the geometrical fractal structures, as the quantitative distribution in subclusters of any level may differ from the ideal distribution and, thus, the organism quality changes. Therefore, the following algorithm to determine the highly sensitive criterion of the FC effectiveness was proposed in [4]. The FC dimension of the organism’s resource space (which we further call the D-criterion of the resource distribution effectiveness) is determined with the formula

(8) $$D=\frac{\log\sum_{i_{1}=1}^{5}\sum_{i_{2}=1}^{5}\ldots \sum_{i_{n}=1}^{5}\delta_{i_{1}i_{2}\cdots i_{n}}^{★}}{\log N},$$

where *N* is a total number of clusters and subclusters and the values $\delta_{i_{1}i_{2}\cdots i_{n}}^{★}$ are calculated with the relations

(9) $$\delta_{i_{1}i_{2}\cdots i_{n}}^{★}=1-\frac{\left|C_{i_{1}i_{2}\cdots i_{n}}-{\bar{C}}_{i_{1}i_{2}\cdots i_{n}}^{ideal}\right|}{{\bar{C}}_{i_{1}i_{2}\cdots i_{n}}^{ideal}}.$$

There are the following reasons for the definition of the *D*-criterion presented in [4]: because the resource space is fractal, then, by an analogy with fractal geometry, where the dimension of the space is determined by the Hausdorff dimension criterion, it is logical to introduce the corresponding criterion, where the resource metric is introduced instead of the geometric metric (deviation in the values of the resources in the hierarchical structure of the fractal-cluster matrix of the organism from their reference values). The *D*-criterion is qualitatively different from the Hausdorff dimension criterion: The *D*-criterion, in contrast to the Hausdorff dimension *D*, can take not only integer and fractional values but also negative values. In addition, the *D*-criterion obeys the resource conservation law (2). An approbation of the *D*-criterion shows its high sensitivity compared to the FC entropy H and the *D*-criterion. The efficiency of the resource allocation (the *D*-criterion) of an organism is understood as a deterministic measure of the deviation in the values of its clusters and subclusters from their reference values.

A mixed FC criterion of organisms was presented in [4], as follows:

(10) $$\chi =\frac{H\cdot D\cdot \eta^{\Sigma }}{H_{0}\cdot D^{max}}.$$

**Figure 3:**
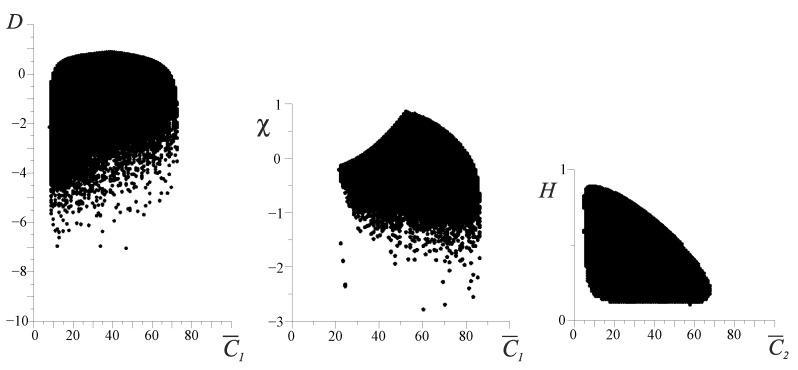
The range of possible values of the *D*, χ and FC entropy *H* for cluster ${\bar{C}}_{1}$. The range of possible states of organisms corresponds to Equation (1). The vertical axes represent values of the *D*-criterion, the mixed χ-criterion and the FC entropy *H*, respectively. The horizontal axes represent values of the energy cluster ${\bar{C}}_{1}$ as a percentage.

**Figure 4:**
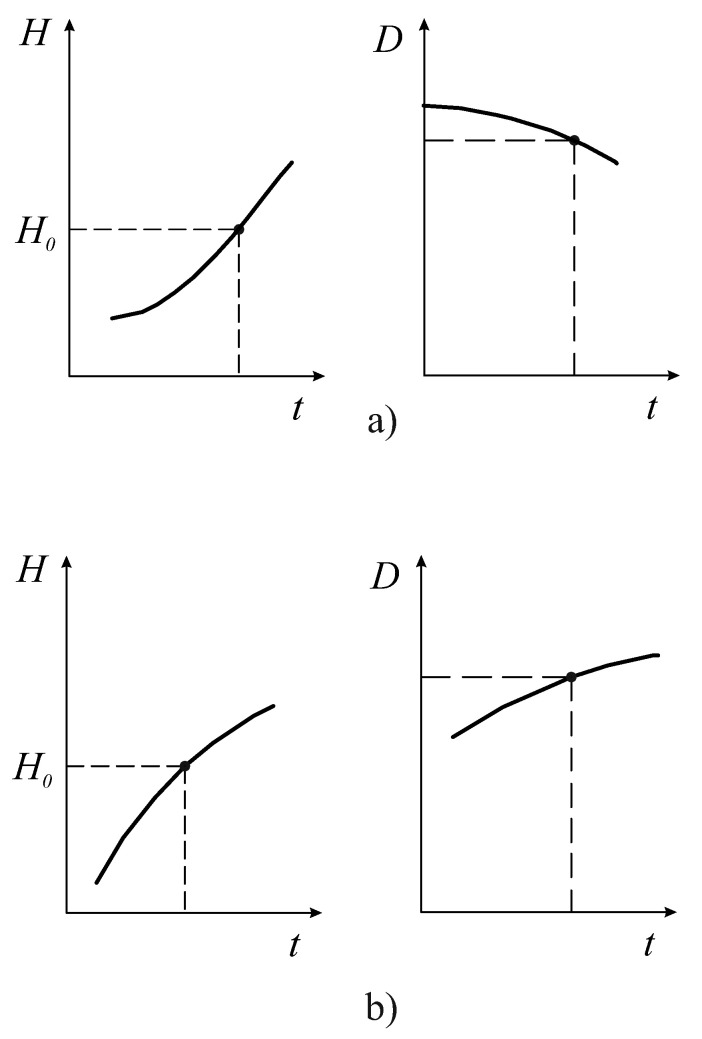
Changing the FC criteria in time: a negative transformation of the organism (**a**); a positive state and transformation of the organism (**b**). The vertical axes represent values of the FC entropy *H* and *D*-criterion, respectively. The horizontal axis represents the time.

Unlike the entropy *H* and full effectiveness $\eta^{\Sigma }$, the free energy *F*, *D*-criterion and χ are very sensitive indicators of varying the FCM values (Figure 3). The area outside the boundary (hatched area) is a nonfunctional state of the organism. Figure 3a shows that in the sector of the negative values of the *D* and χ criteria, at the boundary of the system, we have the destruction of the space continuity where the FCM parameters can vary. This phenomenon can be interpreted as the boundary where irreversible damage of the organism functioning appears. The destruction of the continuity of the phase space of the organism’s resources ($D,C_{1}$) can be interpreted as an FC percolation of the organism’s resources (Figure 3). It is obvious that FC percolation of the resource space of the organism is a catastrophe and is directly related to P. Buck’s theory of self-organizing criticality [9,10]. Figure 4 shows an example of the sufficient and necessary conditions of the resource distribution effectiveness. From these figures, it is clear that the point $H_{0}$ on the entropy trend (on the left in Figure 4a) is the unstable state of the FC resource distribution of the organism, and the point $H_{0}$ on the entropy trend (on the left in Figure 4b) is the stable state of it (see also Section 4).

## 4. Analysis of the Static and Dynamic Stability for Organism’s Evolution

The stability analysis of the biological species’ evolution can be investigated on the basis of fractal-cluster entropy. For this purpose, it is necessary to use the apparatus of I. Prigogine’s thermodynamics of structure [20,21] and nonlinear nonequilibrium fluctuation–dissipation thermodynamics [22]—the minimum entropy production theorem for the states close to the equilibrium state. For the states far from equilibrium, a quadratic oscillating form—a criterion for the production of excess entropy—is used.

We consider clusters $\left\{{\bar{C}}_{i}\right\}$ and subclusters $\left\{{\bar{C}}_{ij}\right\}$, components of the organism, as random internal parameters $C_{i}\left(t\right)$, $C_{ij}\left(t\right)$ changing in a fluctuational manner. If the organism is isolated, the 2-level FC entropy $H_{2}=H_{2}\left(\left\{{\bar{C}}_{ij}\right\}\right)$ does not decrease with time. However, a micro disturbance of the Second Law of thermodynamics for organisms cannot exceed the value of Boltzmann’s constant *k* (see, for example, [22]). This fact allows us to obtain an assessment of the fluctuational component for the fractal-cluster entropy $H_{2}\left(\left\{{\bar{C}}_{ij}\right\}\right)$:

(11) $$\sqrt{{\left(\delta H_{2}\left({\bar{C}}_{ij}\left(t\right)\right)\right)}^{2}}<k^{\prime },$$

where $k^{\prime }$ is a dimensionless analog of Boltzmann’s constant. Let us divide the evolution time into *N*$\left(N\gg 1\right)$ identical intervals Δt. By performing subcluster value averaging on each interval

(12) $$\langle {\bar{C}}_{ij}\rangle \left(t\right)\equiv \frac{1}{\Delta t}\int_{0}^{\Delta t}{\bar{C}}_{ij}\left(t+t^{\prime }\right)dt^{\prime }$$

we go to a new time variable τ:

$$\tau \left(t\right)=\left[\frac{t}{\Delta t}\right]\Delta t,$$

where the symbol $\left[\cdots \right]$ denotes the integer part. Formulas (11) and (12) refer to the estimates of the entropy fluctuations, according to [22], and the averaging of the cluster values over a certain time interval. The FC entropy $H_{2}=H_{2}\left(\tau \left(t\right)\right)$ becomes a step function of *t* and in the case of asymmetric FCMs has the following view:

(13) $$H_{2}\left(\tau \left(t\right)\right)=\langle {\bar{C}}_{1}\rangle +\sum_{j=2}^{5}\langle {\bar{C}}_{1j}\rangle .$$

Formula (13) refers to the determination of the relationships between the clusters and subclusters of a two-dimensional symmetric FCM.

It is assumed that the time interval Δt is much less than evolution time *T* from the initial state $\left\{{\bar{C}}_{ij}^{0}\right\}$ to the final (ideal) organism’s state ${\left\{{\bar{C}}_{ij}\right\}}^{fin\left(ideal\right)}$

(14) $$\Delta t\ll T,\;\langle {\bar{C}}_{ij}^{0}\rangle \;\overset{T}{⟶}\;{\langle {\bar{C}}_{ij}\rangle }^{fin\left(ideal\right)}.$$

In the symmetric case, subclusters $\langle {\bar{C}}_{ij}\rangle$ are determined with the relations

(15) $$\langle {\bar{C}}_{ij}\rangle =\langle {\bar{C}}_{ji}\rangle \;\mathrm{and}\;\langle {\bar{C}}_{ij}\rangle =\langle {\bar{C}}_{i}\rangle \cdot \langle {\bar{C}}_{j}\rangle ,\;i.e.\;\langle {\bar{C}}_{ii}\rangle ={\langle {\bar{C}}_{i}\rangle }^{2}.$$

The FC entropy in this case is

(16) $$H_{2}=2\langle {\bar{C}}_{1}\rangle -{\langle {\bar{C}}_{1}\rangle }^{2}.$$

In accordance with the criterion of thermodynamic stability [21], the second differential of the FC entropy $H_{2}$ for the symmetric case is defined in the following way:

(17) $$\delta^{2}H_{2}=\frac{\partial^{2}H_{2}}{\partial {\langle {\bar{C}}_{1}\rangle }^{2}}{\left(\delta \langle {\bar{C}}_{1}\rangle \right)}^{2}=-2{\left(\delta \langle {\bar{C}}_{1}\rangle \right)}^{2}\leq 0.$$

This formula is a special case of Formula (18) for a symmetric FCM (see below).

Thus, for the states close to thermodynamic equilibrium for the symmetric FCM, the second differential of the entropy $\delta^{2}H_{2}$ is negative, i.e., the organism is stable. The loss of stability for the symmetric FCM is realized only when $\delta \langle {\bar{C}}_{1}\rangle =0$, that is, in the complete absence of fluctuations in energy cluster $\langle {\bar{C}}_{1}\rangle$.

In all other cases of the symmetrical FCM in the states close to the branch of the thermodynamic equilibrium, the stability criterion is satisfied: $\delta^{2}H_{2}<0$.

In the case of the asymmetrical FCM, the second differential of the FC entropy has the form

(18) $$\delta^{2}H_{2}\left(\langle {\bar{C}}_{1}\rangle ,\langle {\bar{C}}_{21}\rangle ,\langle {\bar{C}}_{31}\rangle ,\langle {\bar{C}}_{41}\rangle ,\langle {\bar{C}}_{51}\rangle \right)=\;=\frac{\partial^{2}H}{\partial {\langle {\bar{C}}_{1}\rangle }^{2}}{\left(\delta {\bar{C}}_{1}\right)}^{2}+\sum_{j=2}^{5}\frac{\partial^{2}H_{2}}{\partial {\bar{C}}_{j1}^{2}}{\left(\delta \langle {\bar{C}}_{j1}\rangle \right)}^{2}+\;+2\frac{\partial }{\partial \langle {\bar{C}}_{1}\rangle }\sum_{j=2}^{5}\left(\frac{\partial H_{2}}{\partial \langle {\bar{C}}_{j1}\rangle }\cdot \delta \langle {\bar{C}}_{j1}\rangle \right)\left(\delta \langle {\bar{C}}_{1}\rangle \right)+\;+2\sum_{j=2}^{5}\sum_{j>i}^{5}\frac{\partial^{2}H_{2}}{\partial \langle {\bar{C}}_{i1}\rangle \partial \langle {\bar{C}}_{j1}\rangle }\delta \langle {\bar{C}}_{i1}\rangle \delta \langle {\bar{C}}_{j1}\rangle .$$

In the case of the independence of the energy cluster $\langle {\bar{C}}_{1}\rangle$ and energy subclusters $\langle {\bar{C}}_{21}\rangle ,$ $\langle {\bar{C}}_{31}\rangle ,$$\langle {\bar{C}}_{41}\rangle$ and $\langle {\bar{C}}_{51}\rangle$, the second differential of the FC entropy $\delta^{2}H_{2}$ is determined as follows:

(19) $$\delta^{2}H_{2}=0,$$

that is, even in the presence of fluctuations, neutral stability of the organism’s evolution occurs. In the case of the linear dependence of the energy cluster $\langle {\bar{C}}_{1}\rangle$ and energy subclusters $\langle {\bar{C}}_{21}\rangle ,$ $\langle {\bar{C}}_{31}\rangle ,$$\langle {\bar{C}}_{41}\rangle ,$ $\langle {\bar{C}}_{51}\rangle$, neutral stability also occurs. In the case of the nonlinear dependence of subclusters $\left\{\langle {\bar{C}}_{ij}\rangle \right\}\;\left(i>1\right)$ from the energy cluster, there may appear both stable and unstable regimes of the FCM evolution, i.e.,

(20) $$\delta^{2}H_{2}\left\{\begin{array}{c} <0-\mathrm{stable}\;\;\mathrm{regime},\\ =0-\mathrm{neutral}\;\;\mathrm{stability},\\ >0-\mathrm{unstable}\;\;\mathrm{regime}. \end{array}\right.$$

The system (20) defines the conditions of stability according to Prigogine’s theory [21].

The analysis of the organism’s stability given above which is based on generalized thermodynamics of irreversible processes (Prigogine [20,21]) and the proposed FC theory applies to the states, close to the thermodynamic equilibrium branch, that is, to the linear thermodynamics of irreversible processes. The criterion of stability for organisms, relevant to the concept of “dissipative structures” given by Prigogine, is a quadratic oscillating form, called the excess entropy production [21]. For the stable dissipative structures, the excess entropy production is a positively definite value

(21) $$P\left[\delta^{2}H\right]\equiv \frac{1}{2}\frac{d}{dt}\left(\delta^{2}H\right)>0,$$

where $H=H\left(t\right)$ is the entropy of a general organism. Formula (21) determines the expression of the criterion of organism stability (the excess entropy production) located far from the state of equilibrium [21].

As it has been noted in [20], in a general case, the sign of excess entropy production cannot be determined unequivocally. To determine the $P\left[\delta H\right]$ sign, the use of phenomenological laws is required.

For the FC description of an organism’s resource structure, located far from equilibrium, the following expression for the quadratic oscillating form is obtained, i.e., for the production of excess entropy (or quasi-entropy) for the symmetric case of FCM $\left({\bar{C}}_{ij}={\bar{C}}_{ji}\right)$,

(22) $$P\left[\delta^{2}H_{2}\right]=-\frac{{\left[\delta \langle {\bar{C}}_{1}\rangle \left(t+\Delta t\right)\right]}^{2}-{\left[\delta \langle {\bar{C}}_{1}\rangle \left(t\right)\right]}^{2}}{\Delta t}.$$

The sign of the oscillating form $P\left[\delta^{2}H_{2}\right]$ is determined from the sign of the right-hand side of Equation (22). Thus, we can conclude that the synthesis of the FC theory and generalized thermodynamics of irreversible processes allows us to determine the type of stability criterion for organisms placed far from the equilibrium state.

## 5. Stochastic Distributions in the Fractal-Cluster Theory

Based on the fractal-cluster deterministic criteria (Secton Section 3), we can receive the criterion estimations about the efficiency of the organism’s functioning. But the real organism has a stochastic behavior. Nevertheless, there is a connection between the deterministic criteria and the probabilistic performances of the organism.

Based on the possible values of the cluster combinations and the conservation law (2), the maximal and minimal values of the clusters $\left\{C_{i}\right\}$ are determined:

(23) $$\alpha_{i}\leq C_{i}\leq \beta_{i},\;i=1÷5,\;\left\{\alpha_{i}\right\}=\left\{\begin{array}{c} 0.078\\ 0.063\\ 0.035\\ 0.03\\ 0.02 \end{array}\right\},\;\left\{\beta_{i}\right\}=\left\{\begin{array}{c} 0.7\\ 0.67\\ 0.63\\ 0.6\\ 0.55 \end{array}\right\}$$

For every interval of the allowed cluster values, we can divide these intervals into *n* shares for each cluster. The possibility of the organism’s functioning is realized under the execution of the conservation law (2).

**Figure 5:**
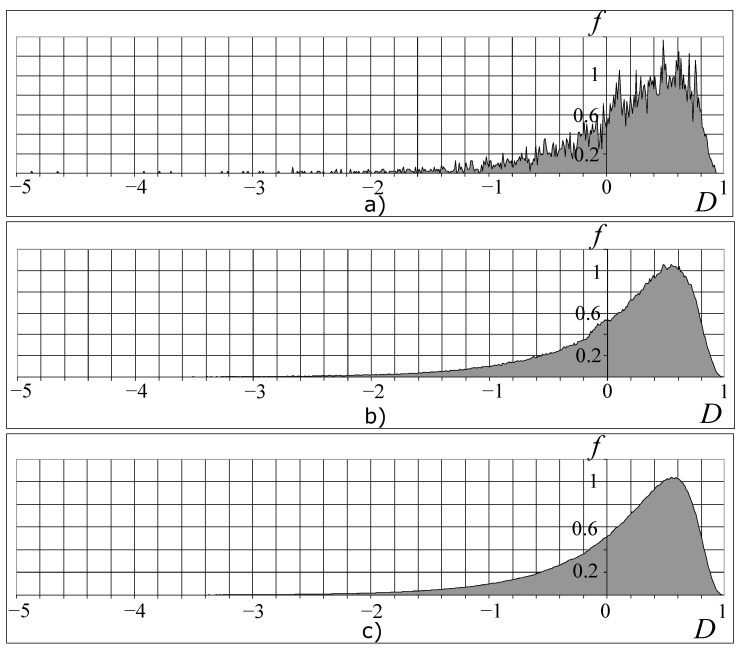
Dependence of the FC density of probability on the *D*-criterion for a different number *n* of partitioning of cluster intervals: n=30 (**a**), n=50 (**b**) and n=100 (**c**). The vertical axis shows values of the FC density of probability *f*, and the horizontal axis shows values of the *D*-criterion.

**Figure 6:**
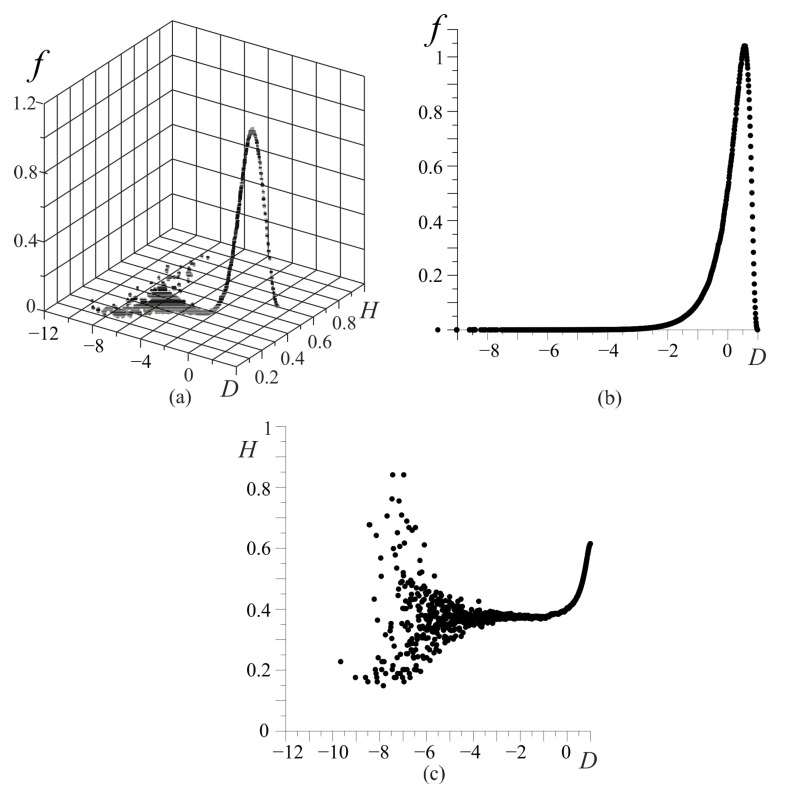
Stochastic characteristics of the FC system states: the dependence of the FC probability density f(D,H) (**a**); the dependence of the FC probability density f(D) (**b**); the dependence $H\left(D\right)$ (**c**). The vertical axis represents the FC density of probability *f*, and the horizontal axes represent values of the *D*-criterion and FC entropy *H*, respectively (**a**); the vertical axis represents the FC density of probability *f*, and the horizontal axis represents the values of the *D*-criterion (**b**); the vertical axis represents the FC entropy *H*, and the horizontal axis shows values of the *D*-criterion (**c**).

That is why only some combinations of the valid values clusters $\left\{C_{i}\right\}$ of the organism can be realized. Figure 5 presents the distributions of the possible organism states under the various divisions of the valid cluster intervals. As shown in Figure 5, beginning with n>50 (*n* is a number of the cluster-dividing interval), the picture of these distributions becomes constant. We can also see the fractal-cluster distribution of the probability density as a function of the *D* and *H* criteria (Figure 5 and Figure 6a–c). The curve cone of the fractal-cluster states looks like the “Golden Shofar” [18,19]. In the author’s opinion, this similarity between the illustration of the fractal-cluster theory and the topology of the mathematical theory of the “Golden Ratio” [18,19] has a deep correlation.

The obtained distribution (Figure 6a) cannot be described with the well-known stochastic distributions. In the phase fractal-cluster space, each value of the *D*-criterion (Figure 6b) corresponds to an essential number of the cluster’s combinations which have to satisfy the resource conservation law (2).

An approximation of the dependence P−D (Figure 6b) has the following view:

(24) $$P\left(D\right)=\frac{a+b\cdot D+c\cdot D^{2}}{h+f\cdot D+g\cdot D^{2}}\cdot \frac{{\left(1-D\right)}^{d}}{c_{1}\left(\exp\left(\frac{1-D}{c^{2}}\right)-1\right)},$$

where a=−9.391, b=8.308, c=0.705, f=−5.776, g=3.474, h=2.617, $H_{0}=0.618$, $d=4H_{0}$, $c_{1}=-0.592$, $c_{2}=\frac{H_{0}}{4\left(1-\frac{H_{0}}{50}\right)}$.

Each cluster combination is equally possible under a fixed value of the *D*-criterion (Figure 6b). To reduce the number of cluster combinations, we can use the thermodynamic method of the free energy minimum principle for each value of the *D*-criterion. This gives a decrease in the intervals’ number with the valid cluster values and this is very important to predict the functioning of real organisms. Reducing the number of cluster intervals by account of the thermodynamic method allows us to use the stochastic method.

## 6. Discussion and Summary

**Figure 7:**
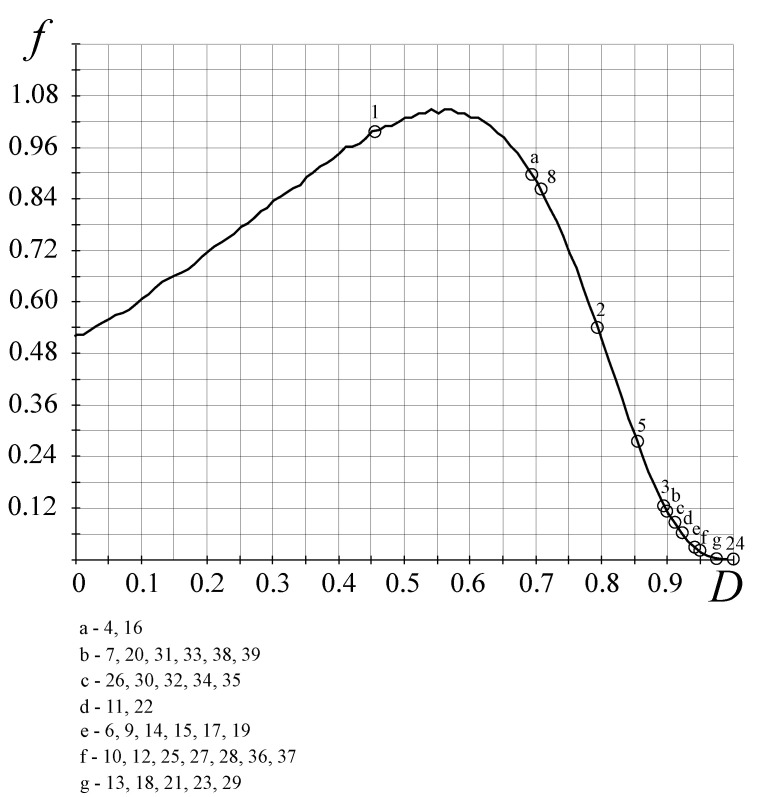
Stochastic FC law for the appearance of biological organisms. The circle numbers in the figure correspond to the characteristics of the organisms in Table 3 and Table 4. The vertical axis represents the FC density of probability *f* and the horizontal axis shows the values of the *D*-criterion.

**Figure 8:**
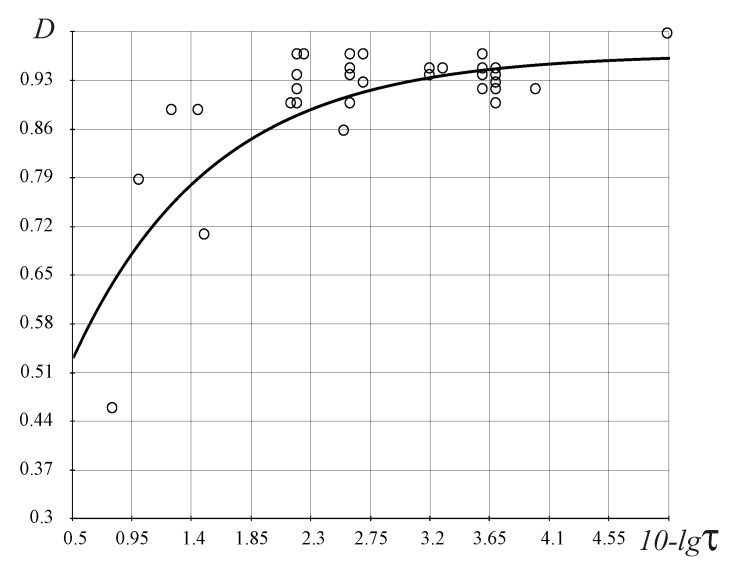
Evolutionary FC law for the biological organisms, $D\left(\tau \right)\approx 1-\frac{H_{0}}{2}t^{-\frac{1}{H_{0}}}$, where t=10−lgτ, $H_{0}=0.618$, and τ is the time of occurrence of organisms in the past (see, for example, refs. [27,28,29,30] and references therein). The vertical axis represents values of the *D*-criterion and the horizontal axis represents the function depending on time t=10−lgτ. Circles in the figure correspond to different organisms in Table 4.

**Figure 9:**
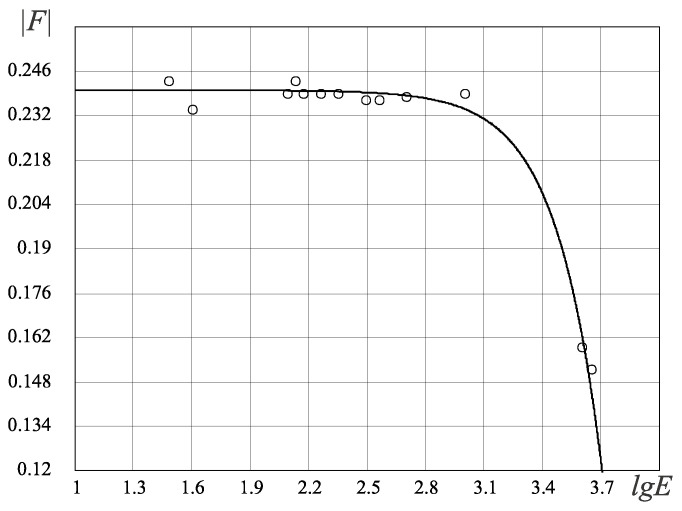
Energy FC law for the biological organisms, $F\left(E\right)\approx 0.24-\frac{E^{3H_{0}}}{H_{0}\cdot 10^{8}}$, $H_{0}=0.618$. The vertical axis represents absolute values $\left|F\right|$ of the *F*-criterion and the horizontal axis represents values of the log power consumption (lgE). Circles in the figure correspond to different organisms in Table 4.

Testing the FC theory for the biological organisms’ research was realized on the basis of study [1,2] (Table 3). In the result of the analysis based on the FC approach, three fundamental FC laws for biological organisms were identified (see also [23], Table 4):

(1) The FC stochastic law defines the density of the probability of the occurrence of biological organisms depending on the perfection of the resource allocation in the organism—the *D*-criterion (Figure 7) shows that there is a bijection and the simpler biological organisms (the *D*-criterion less than perfect), Chlamidomonada and Hydra, have the highest density of probability of its appearance;
(2) The FC evolutionary law (Figure 8) illustrates the increasing complexity and perfection of the emerging organisms through time, from the simpler organisms to humans;
(3) The FC power law (Figure 9) characterizes the energy perfection of biological organisms (the dependence of FC entropy or the *F*-criterion of the energy consumption per 1 kg of organism’s weight per day).

**Figure 10:**
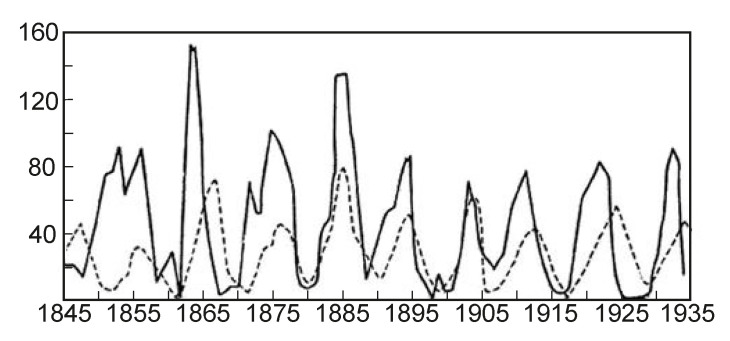
Dynamics of populations of lynxes and hares (the solid line is the hare population, the hatched line is the population of lynxes).

The stochastic law of development of biological organisms was obtained due to the distribution of possible states based on the *D*-criterion and correlates this distribution with the corresponding values of the *D*-criterion for biological organisms (Table 4). The second FC law shows the growth of perfection of the resource distribution in organisms (the *D*-criterion) over time (Figure 8). Figure 9 shows the third revealed FC law that defines a link between the FC free energy of biological organisms and the level of energy consumption per 1 kg of weight. This law represents, in a certain sense, the thermodynamic response to the solution of Lotka–Volterra’s problem “predator-prey” judging by the level of the FC free energy (*F*) of biological organisms (including mammals, fish, insects, etc., behind except for worms the (the last right circles in Figure 9, which have on average the same value of free FC energy, i.e., there is an energy balance between “predators” and their prey—the “predator” cannot catch the “prey”. The FC law of free energy for biological organisms (Figure 9) being thermodynamic confirmation of the solution of the “predator-prey” problem, says that only the “victim” having a level of free energy lower than its average in the population will die. It can be summarized that the decision of the “pendulum” of Lotka--Volterra (Figure 10) is a dynamic energy condition of the impossibility of the destruction of the “victims” by the “predators” and the FC energy law is the static energy condition of that one. It is important to note that the mathematical expression for the second and third laws has the same view:

$$Z\left(x\right)=a-bx^{\alpha },$$

where *a*, *b* and α are constants (Figure 8 and Figure 9).

The review of biophysics studies (see Section 1) shows that the current stage of the convergence of biology and physics reflects an increase in the number of publications which use the apparatus of nonequilibrium thermodynamics of Prigogine. As shown, there is research which goes beyond the traditional approach to this issue, for example, Burdakov’s study may be related to this research. Based on Burdakov’s research [1] for organisms and the analytical apparatus of Prigogine’s theory [20], the foundations of the fractal-cluster theory for the analysis of organisms were presented. As is known, Haken’s approach allows us to describe the organism’s trajectory in the phase space at a known value of the “order parameter”, which is determined from the experiment. Prigogine’s approach does not need experimental data for the description of an organism’s functioning as it has the universal thermodynamic tools at its basis. But Prigogine’s approach cannot describe the trajectory of an organism. This article suggests a new alternative approach to the systematization and study of the evolution of biological organisms on the basis of the fractal-cluster theory. This theory, in a certain sense, presents the compromise between Prigogine’s and Haken’s approaches [24] in synergetics and allows us to describe the organism phase trajectory in the $5^{n}$-dimensional fractal-cluster space without additional experimental information. It presents the criterion’s apparatus of the resource distribution in organisms and the stochastic distributions for the organism states in the $5^{n}$-dimensional FC space.

This article gives a definition of the organism in the fractal-cluster approach which is qualitatively different from the one generally accepted in biology and biophysics. But, at the same time, it shows the relationship of seven classic factors of the organism definition [15] and the definition of the organism with the FC approach. This article also proves that von Stockar’s findings [15,25,26] of the optimal growth efficiency for organisms have a connection with the fractal-cluster theory. In agreement with the FC theory, the free energy is determined as follows:

$$F=C_{1}-H,$$

where $C_{1}$ is the energy cluster value and *H* is the fractal-cluster entropy. For the two-dimensional symmetric FC matrix, the free energy *F* is equal $F=-C_{1}+C_{1}^{2}$. The maximal absolute value of the free energy is defined as follows:

$$\frac{dF}{dC_{1}}=0,\;C_{1}=0.5,\;{\left|F\right|}_{\max}=0.25.$$

The optimal value of the free energy corresponds to the ideal (standard) values of clusters and is defined as follows:

$$C_{1}^{\left(\mathrm{ideal}\right)}=0.38,\;{\left|F\right|}_{\mathrm{opt}}=0.235.$$

Thus, the optimal value of the free energy is 94% from the maximal value of the one:

$${\left|F\right|}_{\mathrm{opt}}=0.94{\left|F\right|}_{\max}.$$

Ergo, the share of free energy going to the cell growth will be smaller than $F_{\mathrm{opt}}$: $F_{\mathrm{grows}}<F_{\mathrm{opt}}$, i.e., 100% smaller. This fact qualitatively and quantitatively confirms von Stockar’s result by FC theory. As a sample of the FC tool used for the organism’s analysis, the FC criterion estimation for the systematization of biological organisms has been presented. The conducted criterion analysis of 39 species of biological organisms allowed us to identify three fundamental FC laws: (1) the FC stochastic law; (2) the FC evolutionary law; and (3) the FC energy law. The probabilistic law connects the thermodynamic perfection of the organism (the *D*-criterion) with the fractal-cluster density of probability and the time of the appearance of the organism species: the simpler FC organisms (the *D*-criterion is less) have a greater likelihood of their occurrence. The evolutionary law connects the thermodynamic perfection of the organism with the time of its occurrence.

The fractal-cluster theory is an additional toolkit to the generally accepted theory of the evolution of organisms, which makes it possible to give an exclusively thermodynamic assessment of their development and functioning. In particular, an alternative solution to the famous Volterra–Lotka’s problem is presented for the first time on the basis of fractal-cluster criteria—a static energy assessment was obtained about the impossibility of the complete destruction of the “game” by the “predator”—in contrast to the known dynamic solution of this problem. It is doubtless that the new analytical apparatus allows us to obtain new knowledge about the object under study.

As for the prospects of applications of the fractal-cluster theory in biology, they can probably be implemented in cell technologies, in virology, as well as in medico-biological applications and for the ecological system’s analysis [31]. The impact of the external environment on the organism leads to a redistribution of the organism’s resources, which in turn makes it possible to use the fractal-cluster criteria, and as a result, to obtain characteristics of the stability of the organism’s functioning, an opportunity to obtain information in advance about the pathological trends of its development.

## Figures and Tables

**Table 1:** Basic characteristics of biological organisms when modeled by traditional methods and tools of the fractal-cluster theory.

	Traditional Description of Biological Organisms	FC Description of Biological Organisms
Complex organisms, open system	Yes	Yes
Biochemical, biophysical machines	Yes	Do not contradict
Reacts as a whole to external influences	Yes	Yes
Hierarchy of organisms	Yes	Yes, except for the level
Reproduction	Yes	Does not contradict
Transmission of hereditary information	Yes	No
Variability	Yes	Does not contradict
Individual development	Yes	Yes
Evolution of development	Yes	Yes
Rhythmicity	Yes	Yes
The possibility of studying FC tools	No	Yes
Identification of the organism as self-organizing system independently satisfying basic needs	No	Yes

**Table 2:** Table of the ideal cluster values.

*j*	${\bar{C}}_{j}^{\mathrm{ideal}}$	${\bar{C}}_{1j}^{\mathrm{ideal}}$	${\bar{C}}_{2j}^{\mathrm{ideal}}$	${\bar{C}}_{3j}^{\mathrm{ideal}}$	${\bar{C}}_{4j}^{\mathrm{ideal}}$	${\bar{C}}_{5j}^{\mathrm{ideal}}$
1	0.38	0.144	0.1026	0.0608	0.0494	0.0228
2	0.27	0.1026	0.0729	0.0432	0.0351	0.0162
3	0.16	0.0608	0.0432	0.0256	0.0208	0.096
4	0.13	0.0494	0.0351	0.208	0.0169	0.078
5	0.06	0.0228	0.0169	0.096	0.078	0.0036

**Table 3:** Cluster values for the biological organisms (males).

	Organism’s Names	Mass, kg	FCR, %				
			$C_{1}$	$C_{2}$	$C_{3}$	$C_{4}$	$C_{5}$
1	Chlamidomonas	$3\cdot 10^{-11}$	40 ± 10	10 ± 8	30 ± 6	10 ± 5	10 ± 5
2	Hydra vulgaris	$10^{-5}$	40 ± 10	30 ± 8	10 ± 6	10 ± 5	10 ± 5
3	Scorpiones mingrelicus	$5\cdot 10^{-4}$	33 ± 6	33 ± 5	17 ± 4	9 ± 3	6 ± 2
4	Oligochaeta	$10^{-3}$	19 ± 8	50 ± 10	16 ± 4	10 ± 3	5 ± 2
5	Anisoptera libellula depressa	$10^{-3}$	40 ± 8	23 ± 5	17 ± 4	10 ± 3	10 ± 2
6	Micromys minitus	$5\cdot 10^{-3}$	40 ± 6	27 ± 5	16 ± 4	10 ± 3	7 ± 2
7	Rona ridibunda	0.05	40 ± 5	30 ± 4	16 ± 3	8 ± 2	6 ± 1
8	Testudo horsefieldi	0.1	38 ± 6	20 ± 5	30 ± 4	7 ± 3	5 ± 1
9	Cucules canorus	0.1	40 ± 6	27 ± 5	16 ± 4	10 ± 3	7 ± 2
10	Procellariida	0.8	40 ± 6	28 ± 5	16 ± 4	10 ± 3	6 ± 2
11	Larus argentatus	1.0	39 ± 6	28 ± 5	16 ± 4	10 ± 3	7 ± 2
12	Heroestes edwardsi	3.0	40 ± 6	27 ± 5	17 ± 4	10 ± 3	6 ± 2
13	Ciconia ciconia	4.0	40 ± 6	27 ± 5	16 ± 4	11 ± 3	6 ± 2
14	Lepus timidus	5.0	40 ± 6	28 ± 5	16 ± 4	11 ± 3	5 ± 2
15	Grus grus	6.0	40 ± 6	27 ± 5	16 ± 4	10 ± 3	7 ± 2
16	Paralithodes camtchatica	7.0	20 ± 6	50 ± 5	16 ± 4	8 ± 3	6 ± 2
17	Pelecanida onocrotalus	10	40 ± 6	28 ± 5	16 ± 4	11 ± 3	5 ± 2
18	Vulpes	10	40 ± 6	27 ± 5	16 ± 4	11 ± 3	6 ± 2
19	Castor fiber	30	40 ± 6	26 ± 5	17 ± 4	12 ± 3	5 ± 2
20	Acinonyx jubatus	50	40 ± 6	30 ± 5	16 ± 4	8 ± 3	6 ± 2
21	Canis lipus	50	40 ± 6	27 ± 5	16 ± 3	11 ± 3	6 ± 2
22	Pan troglodytes	60	39 ± 6	28 ± 5	16 ± 3	11 ± 2	6 ± 2
23	Orycturopus afer	70	40 ± 6	28 ± 5	17 ± 3	11 ± 2	5 ± 2
24	Homo sapiens	75	38 ± 6	27 ± 5	16 ± 4	13 ± 2	6 ± 1
25	Ursus arctos	100	40 ± 6	27 ± 5	17 ± 4	10 ± 3	6 ± 2
26	Cervina nippon	120	40 ± 6	28 ± 5	17 ± 4	9 ± 3	6 ± 2
27	Sus scrofa	150	40 ± 6	28 ± 5	16 ± 4	10 ± 3	6 ± 2
28	Pongo pygmaeus	200	39 ± 6	29 ± 5	16 ± 3	10 ± 3	6 ± 2
29	Gorilla gorilla	250	39 ± 6	28 ± 5	16 ± 3	11 ± 2	6 ± 1
30	Equida burchelli	300	40 ± 7	28 ± 6	17 ± 4	9 ± 3	6 ± 2
31	Tursiops	400	42 ± 7	28 ± 6	16 ± 4	8 ± 3	6 ± 2
32	Equus caballus	400	40 ± 7	28 ± 6	17 ± 4	9 ± 3	6 ± 2
33	Galeocerdo cuvieri	500	40 ± 6	30 ± 5	16 ± 4	8 ± 3	6 ± 2
34	Camelus bactrianus	600	40 ± 6	27 ± 5	18 ± 4	9 ± 3	6 ± 2
35	Giraffa cameleopardalis	750	40 ± 6	29 ± 5	16 ± 4	10 ± 3	5 ± 2
36	Hippopotamus amphibius	3000	40 ± 6	28 ± 5	16 ± 4	10 ± 3	6 ± 2
37	Loxodonta africana	5000	40 ± 6	28 ± 5	16 ± 4	10 ± 3	6 ± 2
38	Balaena mysticetus	150,000	42 ± 5	28 ± 4	16 ± 3	8 ± 2	6 ± 1
39	Balaenoptera musculus	200,000	42 ± 5	28 ± 4	16 ± 3	8 ± 2	6 ± 1

**Table 4:** FC criterion estimation for the biological organisms.

	Organism’s Names	*D*	*H*	$C_{1}$	*F*
1	Chlamidomonas	0.46	0.622	0.387	−0.235
2	Hydra vulgaris	0.79	0.637	0.4	−0.237
3	Scorpiones mingrelicus	0.89	0.561	0.338	−0.223
4	Oligochaeta	0.69	0.342	0.19	−0.152
5	Anisoptera libellula depressa	0.86	0.832	0.4	−0.238
6	Micromys minitus	0.94	0.639	0.4	−0.239
7	Rona ridibunda	0.90	0.639	0.4	−0.239
8	Testudo horsefieldi	0.71	0.614	0.38	−0.234
9	Cucules canorus	0.94	0.639	0.4	−0.239
10	Procellariida	0.95	0.662	0.42	−0.242
11	Larus argentatus	0.93	0.627	0.39	−0.237
12	Heroestes edwardsi	0.95	0.639	0.4	−0.239
13	Ciconia ciconia	0.97	0.664	0.421	−0.243
14	Lepus timidus	0.94	0.639	0.4	−0.239
15	Grus grus	0.94	0.664	0.421	−0.243
16	Paralithodes camtchatica	0.69	0.359	0.2	−0.159
17	Pelecanida onocrotalus	0.94	0.639	0.4	−0.239
18	Vulpes	0.97	0.639	0.4	−0.239
19	Castor fiber	0.94	0.664	0.421	−0.243
20	Acinonyx jubatus	0.90	0.639	0.4	−0.239
21	Canis lipus	0.97	0.639	0.4	−0.239
22	Pan troglodytes	0.93	0.627	0.39	−0.237
23	Orycturopus afer	0.97	0.633	0.395	−0.238
24	Homo sapiens	1.0	0.614	0.38	−0.234
25	Ursus arctos	0.95	0.639	0.4	−0.239
26	Cervina nippon	0.92	0.639	0.4	−0.239
27	Sus scrofa	0.95	0.639	0.4	−0.239
28	Pongo pygmaeus	0.95	0.627	0.39	−0.237
29	Gorilla gorilla	0.97	0.627	0.39	−0.237
30	Equida burchelli	0.92	0.636	0.4	−0.239
31	Tursiops	0.90	0.659	0.42	−0.242
32	Equus caballus	0.92	0.636	0.4	−0.239
33	Galeocerdo cuvieri	0.90	0.639	0.4	−0.239
34	Camelus bactrianus	0.92	0.639	0.4	−0.239
35	Giraffa cameleopardalis	0.92	0.639	0.4	−0.239
36	Hippopotamus amphibius	0.95	0.639	0.4	−0.239
37	Loxodonta africana	0.95	0.639	0.4	−0.239
38	Balaena mysticetus	0.90	0.663	0.42	−0.243
39	Balaenoptera musculus	0.90	0.663	0.42	−0.243

## Data Availability

The data supporting the findings of this study are available within the article. All other relevant source data are available from the corresponding author upon reasonable request.
